# Minimum Detectable Air Velocity by Thermal Flow Sensors

**DOI:** 10.3390/s130810944

**Published:** 2013-08-19

**Authors:** Safir Issa, Walter Lang

**Affiliations:** IMSAS (Institute for Microsensors, Actuators and Systems), Microsystems Center Bremen (MCB), University of Bremen, Otto-Hahn-Allee, Bld. NW1, Bremen D-28359, Germany; E-Mail: wlang@imsas.uni-bremen.de

**Keywords:** minimum detectable flow, natural convection, noise, mixed convection region, thermal flow sensors

## Abstract

Miniaturized thermal flow sensors have opened the doors for a large variety of new applications due to their small size, high sensitivity and low power consumption. Theoretically, very small detection limits of air velocity of some micrometers per second are achievable. However, the superimposed free convection is the main obstacle which prevents reaching these expected limits. Furthermore, experimental investigations are an additional challenge since it is difficult to generate very low flows. In this paper, we introduce a physical method, capable of generating very low flow values in the mixed convection region. Additionally, we present the sensor characteristic curves at the zero flow case and in the mixed convection region. Results show that the estimated minimum detectable air velocity by the presented method is 0.8 mm/s. The equivalent air velocity to the noise level of the sensor at the zero flow case is about 0.13 mm/s.

## 1. Introduction

The minimum detectable flow (MDF) becomes a crucial feature when flow sensors are used in very low-flow applications, such as gas detection and accurate supply of gases in some medical applications [1,2]. MDF is the minimum flow passing through the sensor which will give a signal different from noise. It represents a threshold that the flow should exceed to be considered non-zero. This parameter differs from the resolution of the sensor which is defined as the smallest change in a measured quantity which causes a perceptible change in the corresponding indication [3].

MDF is basically influenced by natural (free) convection and thermal noise in the case of thermal flow sensors. Natural convection is a complex mechanism in which the fluid motion is generated by density differences in the fluid due to temperature gradients [4]. The air surrounding the sensor heater receives heat, expands and rises up. The cooler air subsequently moves to replace it. This cooler air is then heated and the process continues, forming convection current. Thermal noise is an electrical noise source caused by random motion of electrical charges in the material.

Flow is characterized mainly by Reynolds number (Re). Re is a dimensionless number used in fluid mechanics to study the flow; it represents the ratio of inertial forces to viscous forces in the fluid. Reynolds number characterizes different flow regimes, *i.e.*, laminar and turbulent flow. Laminar flow is characterized by a low Reynolds number where viscous forces are dominant and fluid flows in parallel layers with no mixing between the layers. By contrast, turbulent flow occurs when inertial forces are dominant and it is characterized by high Reynolds numbers where eddies, vortices, and other flow instabilities are produced. Reynolds number is given by the following equation:

(1) Re=v×L/ν

where v is air velocity; L is the characteristic length and &upsilon; is the kinematic viscosity of the air.

Free convection is characterized by Grashof number (Gr) which expresses the ratio between buoyancy forces due to spatial variation in fluid density (caused by temperature differences) to viscous forces acting on the fluid. It is given as:

(2) $$\text{Gr}=\frac{\text{g}\beta ({\text{T}}_{\text{s}}-{\text{T}}_{\infty }){\text{L}}^{3}}{\nu^{2}}$$

where g is the local acceleration due to gravity; β is the volumetric thermal expansion coefficient (for an ideal gas, β equals the inverse of the absolute temperature); T_s_ and T_∞_ are temperatures of the surface and the surrounding fluid, respectively; L is the characteristic length and &upsilon; is the kinematic viscosity of the fluid. Free convection on a surface depends on several parameters such as geometry, orientation, variation of temperature on the surface and thermo-physical properties of the fluid. For a vertical plate position, the plate is aligned with the gravitational vector, and the buoyancy force induces fluid motion in the upward (or downward) direction. However, if the hot plate is horizontal, as in our case, the buoyancy force is normal to the surface and the resulting fluid motion is in the vertical direction. When the temperature difference (T_s_ − T_∞_) rises, the surrounding air starts to move and the heat losses rise quickly. However, when the convective flow is established, the heat transfer rises slightly with increasing temperature difference [5].

**Figure 1.**
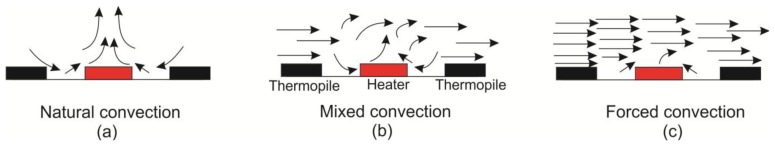
Representation of natural, mixed, and forced convections around thermal flow sensor.

The ratio Gr/Re^2^ defines the importance of natural convection in respect to a forced convection. This ratio of the buoyancy forces and the inertial forces is expressed as:

(3) $$\frac{\text{Gr}}{{\text{Re}}^{2}}=\frac{\text{gL}\beta \Delta \text{T}}{{\text{v}}^{2}}$$

where g is the local acceleration due to gravity; L is the characteristic length of the hot plate; β is the volumetric thermal expansion coefficient, for an ideal gas β equals inverse of the absolute temperature; ∆T is the temperature difference between the heater and surrounding air; and v is the velocity. It is well established that natural convection is negligible when Gr/Re^2^ ≪ 1, forced convection is negligible when Gr/Re^2^ ≫ 1, and both are significant when Gr/Re^2^ ≅ 1. In the strict sense, a free convection flow is induced by buoyancy forces, if there is no well-defined forced convection velocity and Gr/Re^2^ = ∞ [4]. In the flow sensor case, a pure free convection may occur when the “forced” flow is zero as depicted in Figure 1a. However, by increasing the flow, the forced convection increases as well. The free and forced convections enter the mixed convection region where both of them are significant as in Figure 1b. For higher velocities the contribution of the free convection can be neglected as represented in Figure 1c.

Van Putten *et al.* [6] found that the upper limit on the mixed convective region (which is defined by the ratio Gr/Re^2^) equals to [0.3–0.8] for a horizontal hot plate. The method used to generate velocities in the mixed convection region is based on a vertical piston controlled by a computer. It moves back and forth in order to generate the airflow in two opposite directions. A hardware clock in the engine control unit measures the number of rotations of the engine that moving the piston. Velocities achieved by this method ranged from 1 to 25 mm/s, 1 mm/s was clearly detected whereas velocities below 0.5 mm/s could not be generated in a reliable way. Cubacku *et al.* [7] presented a design of a low power 2D flow sensor. They found that the velocity detection limit is about 5 mm/s, which is in the range of the critical velocity for the transition to the mixed convective flow. Microchannels realized by microfluidic structures were used for measuring very low flow rates as reported by some authors, such as, Buchner *et al.* [8] and Patsis *et al.* [9]. In these two reports water flow was used for the evaluation of the thermal flow sensors. Liao *et al.* [10] reported a minimum detectable airflow velocity of 0.2 mm/s by presenting a novel CMOS micromachined capacitive flow sensor for respiratory monitoring. Resolution is also reported in several flow sensors reports. Some examples of the reported resolution values are: 0.1 mm/s in [11], 2 mm/s in [12], and 0.5 m/s in [13].

The focus of this paper is directed towards the minimum detectable air velocity by calometric thermal flow sensor. Thermal noise and free convection are considered as basic parameters which affect MDF. After a short description of the used sensor, we present a simple experimental method provide very low flow rates. It allows obtaining the characteristic curve in the mixed convection region, up to 20 mm/s. This value is the velocity for the upper limit of the mixed convection region determined by the ratio Gr/Re^2^. Then, a statistical study for sensor output at zero flow is done. From the characteristic curve and noise level at zero flow, we calculate the minimum detectable velocity by this method and the corresponding velocity to noise level at zero flow.

## 2. Description of the Proposed Method

**Figure 2.**
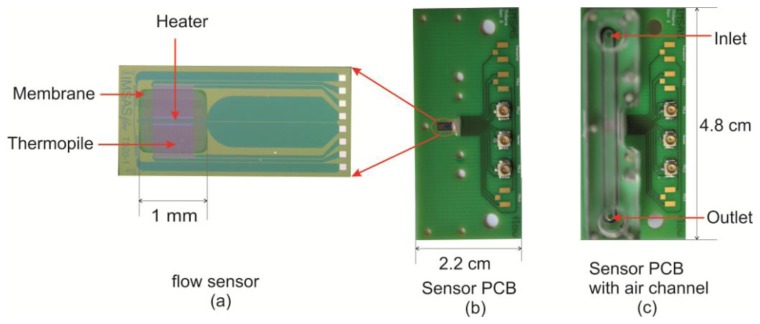
(**a**) IMSAS thermal flow sensor, (**b**) the sensor within its PCB, and (**c**) the air channel mounted on the sensor PCB.

The investigated thermal flow sensor is based on silicon as substrate material; it consists of a heater and two symmetric thermopiles embedded in silicon nitride membrane as shown in Figure 2. The heater is made of tungsten-titanium, whereas the thermopiles are made from a combination of polycrystalline silicon and tungsten-titanium. The sensor membrane area is 1 mm^2^ with a thickness of 600 nm. The distance between heater and both thermopiles is 20 μm. More information about the fabrication process of the sensor can be found in [14]. An airflow channel is mounted on the sensor PCB in such way that the sensor membrane is located in the middle of the channel (see Figure 2). This air channel has rectangular cross section with the dimensions 1.5 × 2 mm^2^. The sensor is operated by a constant power circuit which provides constant power to the heater during the measurement. Response time of the sensor is related to the velocity and geometry of the membrane. It decreases from about 5 ms in the stagnant flow case to 1.5 ms in the case of 44 m/s as air velocity [15,16].

**Figure 3.**
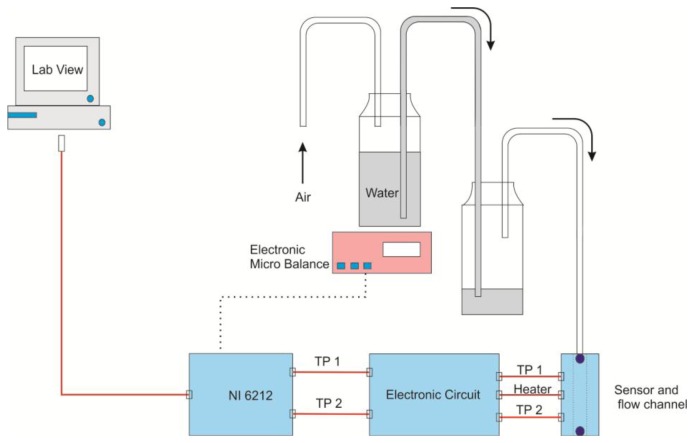
Setup for generating very small flow rates. The flow is identified by measuring the water flow rate between two closed bottles.

In order to measure the sensor MDF, we built a physical method which generates very small flow rates. The method principle is based on weighing the mass changes of one bottle partially filled with water during its discharge into another bottle. Mass readings were taken in time steps of 2.5 s in order to calculate the mass flow. Water flow between the two bottles occurs by means of a small pipe. This method is shown in Figure 3. In the experiments, we initiate a water flow between two bottles, placed in different height positions, by pushing air into the first bottle. This action forces an equivalent air flow to go out from the second bottle. The generated air flow is guided through a pipe to the sensor air-channel. The first bottle is placed on an electronic microbalance (readability 0.1 mg, Sartorius, Göttingen, Germany) interfaced to a computer through DAQ NI 6212 device. Balance readings are synchronized with the sensor output voltage difference through the program LabVIEW. As initial conditions, the first bottle is half full with water and the second one is empty. Then we consider three different cases regarding the height positions of the two bottles. In such a way their height differences are large, moderate and small respectively. In the first two cases, the first bottle is discharged completely into the second one, but at different speeds, whereas in the third case, water starts to flow slowly between the two bottles until equilibrium is reached. This happens when the two bottles have the same water level.

The accuracy of the calculated flow velocity depends basically on the accuracy of the balance. The balance accuracy is 0.1 mg. The mass flow is calculated as successive discrete values. Each one represents the mean flow between two successive weighing operations separated by 2.5 s. Thus we have 600 flow values. The accuracy of velocity values (is calculated by substituting the corresponding values of pipe section area and water density) resulted from using the balance is about 0.03 mm/s. Additionally, the relative errors generated by using the mean velocity value between each two successive weighing operations are less than 5%.

## 3. Results and Discussion

**Figure 4.**
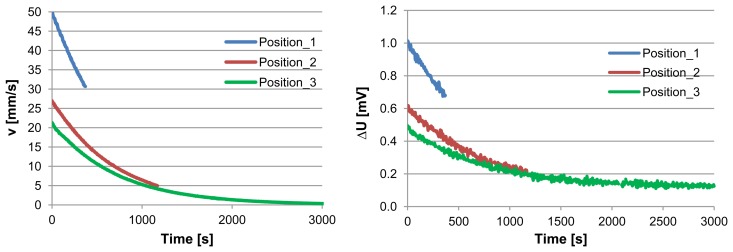
(**left**) Induced air velocities *vs.* time, (**right**) the correspondent sensor output voltage differences *vs.* time for three different positions of both bottles regarding their height difference.

The discharging curve in the previous experiments is exponential as shown in Figure 4. This figure compares the equivalent air velocity (left) and the related sensor output voltage difference (right) *vs.* time. The flow decreases very slowly toward zero. The equivalent air velocity is calculated at 20 °C by assuming that the water density is 998.2 kg/m^3^, and the section area of the air channel is 3 mm^2^. The resulting curve of velocity (v) as function of time has similar behavior of the sensor output voltage difference (∆U) as function of time. In order to obtain the direct relationship between the sensor output voltage difference and air velocity, we modeled the both curves by using MATLAB based program. The resultant fitted curves for the air velocity (in mm/s) and the sensor output voltage difference (in mV), are given in the following expressions, respectively:

(4) $$\text{v}=21.1\times {\text{e}}^{-0.0014\times \text{t}}-0.01$$

(5) $$\Delta \text{U}=0.4\times {\text{e}}^{-0.0014\times \text{t}}+0.12$$

We can obtain the characteristic curve of the sensor in the mixed convention region by eliminating time between the above two equations, which give:

(6) ΔU=0.017×v+0.12

This equation assumes the linear relationship in the mixed convection region. Sensor sensitivity (S) is defined as the derivative of the output voltage difference with respect to the airflow velocity, as in the following equation [17]:

(7) $$\text{S}=\frac{\partial \Delta \text{U}}{\partial \text{v}}$$

**Figure 5.**
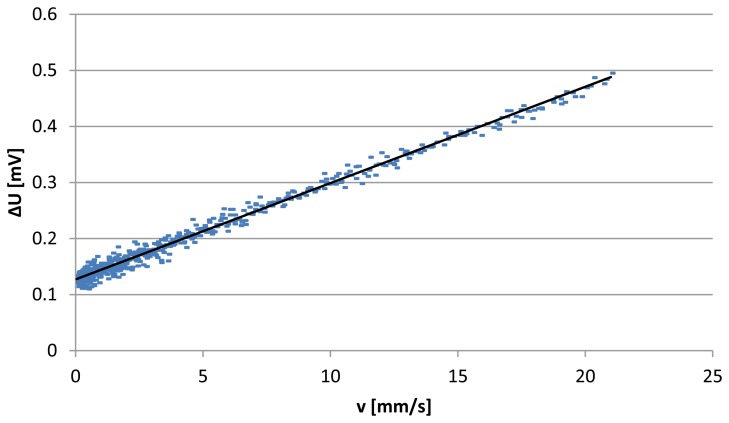
Sensor output voltage difference (∆U) as function of air velocity (v) in the mixed convection region.

The sensitivity is then 0.017 V/m/s. The experimental data between ∆U and v are plotted in Figure 5. This Figure shows their linear relationship and that the number of data in the early part of the curve is very high and then it decreases with the velocity increase. This is due to the constant time step of extracting data. When the two bottles are in the vicinity of equilibrium the flow becomes very slow which causes the accumulation of data in this region. In order to estimate the MDF of the sensor, we calculate first the deviations of all experimental data from the fitted line, by calculating their standard deviation and then divide it by the sensor sensitivity. Standard deviation of the experimental data has been calculated from 600 points, and is 7 μV. We consider 2σ (which represent 95% of the population in a normal distribution). Minimum detectable flow velocity of the sensor by means of this method is then:

(8) $$\text{MDF}=\frac{2\sigma }{\text{S}}=0.8\text{mm}/\text{s}$$

The relative error by this method is less than 20% for the range from 0 to 5 mm/s; it decreases significantly afterwards to less than 10% for the range 5 to 20 mm/s. The larger relative errors for small velocities are due to the high significance of the free convection in heat transfer in addition to the instability of the balance at low values which is another reason for these relatively high errors. By extracting the maximum deviation from the fitting line we found that the maximum error is about 0.03 mV. The corresponding error in velocity according to Equation (6) is about 1.8 mm/s. This MDF result is in the same order as the results found in the previously mentioned reports [6,7]. However, it is higher than the one mentioned in [10] where a different sensor principle is used. Eliminating the error caused by the balance in the previous method is possible by evaluating the sensor noise at zero flow where the natural convection is maximum. This can be done by doing second experiment without flow.

### Zero Flow Case

Noises on the sensor signals are caused by the sensor itself and by the measurement system. At zero flow, new experiments were done to evaluate noise level. The noise of the sensor is mainly caused by thermopiles noises and natural convection. In this case, we can examine the pure natural convection together with the thermal noise as there is no defined forced convection and $\frac{\text{Gr}}{{\text{Re}}^{2}}=\infty$.

Firstly, the thermopile noise is basically the temperature noise and the thermal noise. The temperature noise is caused by temperature fluctuations in the surrounding atmosphere. We assume that this noise has negligible effect on our calculations as all our measurements have been performed at room temperature 20 to 22 °C. Meanwhile the thermal noise or the Johnson noise is an electrical noise source caused by random motion of electrical charges in the material. The Johnson noise is determined by the following equation [18]:

(9) $${\text{V}}_{\text{noise}}=\sqrt{4\times {\text{k}}_{\text{B}}\times {\text{T}}_{\text{ext}}\times {\text{R}}_{\text{e}}\times \Delta \text{f}}$$

where k_B_ is the Boltzmann's constant; T_ext_ is the external temperature; R_e_ is the electrical serial resistance and ∆f is the frequency bandwidth. With k_B_ = 1.38066 × 10^−23^ J/K; T_ext_ = 323 K; R_e_ = 200 K; ∆f = 1 Hz. The thermal noise of the sensor is then 0.06 μV.

**Figure 6.**
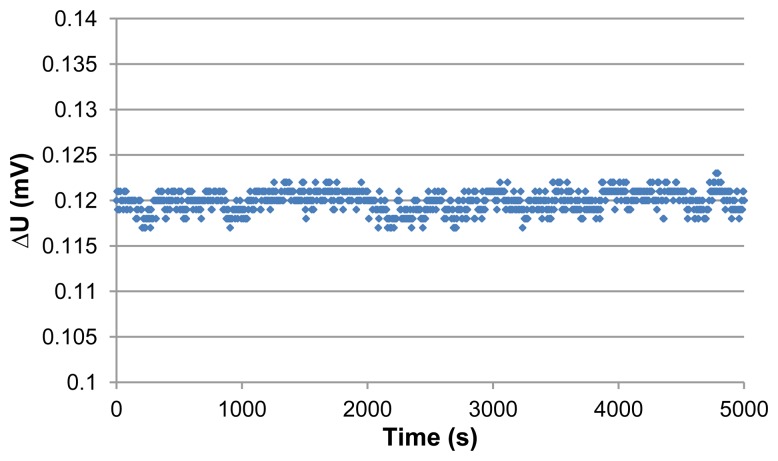
Sensor's output voltage difference *vs.* time in the zero flow case.

**Figure 7.**
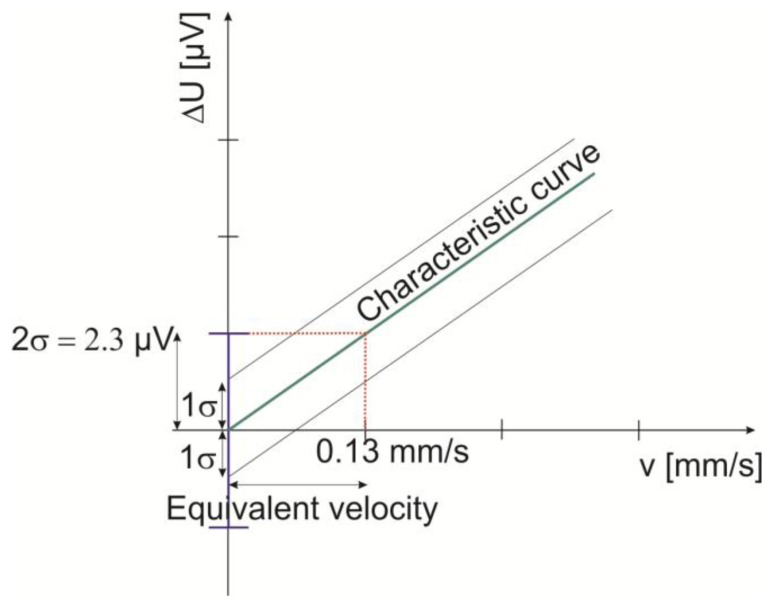
Representation of the detection limit of the flow sensor.

Secondly, the main noise source of the measurement system is that of the Analogue to Digital Convertor (ADC). Since the thermopiles signals are analogue they are converted into digital by ADC with reference voltage of 400 mV and resolution of 16 digits. The root-mean-square quantization noise (N) is obtained from the following equation [19].

(10) $$\text{N}=\frac{\text{q}}{\sqrt{12}}$$

where q equals one Least Significant Bit (LSB). The quantization noise is then 1.76 μV which is much higher than the thermal noise of the sensor. Due to the difficulty of estimating the exact participation of the natural convection and other parameters in the measurement system in the total noise, we performed experimental measurement to identify the noise level at zero flow. The sensor output voltage difference (∆U) at zero flow is extracted for large number of data through a LabVIEW program. The heater was powered on, and then 1,000 samples with time interval step of 5 s were taken. Figure 6 shows the sensor's output voltage difference (∆U) as function of time. The mean value of the extracted data is 0.12 mV which represents the sensor offset, whereas the signal noise expressed as standard deviation (2σ) is about 2.26 μV. The corresponding air velocity to this noise level is calculated according to the Equation (8) and presented in Figure 7:

(11) $$\text{corresponding velocity to the noise level at zero flow}=\frac{2\sigma }{\text{S}}=0.13\text{mm}/\text{s}$$

This value represents the theoretical limit for the minimum detectable flow velocity for the studied sensor.

These results show that thermal flow sensors are capable to detect very low air velocities by optimizing the noise sources. Firstly, the thermal noise of the thermopile is very small as it gives a detection limit of 0.9 μm/s for temperature resolution of 0.1 mK. Secondly, the natural convection can be minimized by either reducing the characteristic length such as by using narrow and deep air channels, or by reducing the temperature difference between the heater and the surrounding air. The first solution requires reducing the sensor dimensions whereas the second solution will decrease the sensor sensitivity and the measuring range. Thirdly, the noise arising from the measurement system can be reduced by optimizing the choices of the circuit elements such as ADC with higher resolution. Moreover, the promising results of using microchannels realized by microfluidic structures in providing very accurate measurements for very low flow rates, especially for liquids, motivate us to use such structure for air as flow as well.

## 4. Conclusion

We have introduced in this paper a physical test method which is capable of generating very low flow values in the mixed convection region from 0 to 20 mm/s. We found that the characteristic curve is linear in this region and the sensor sensitivity is about 0.017 V/m/s. The estimated minimum detectable velocity obtained by the presented method is 0.8 mm/s. Equivalent velocity to the noise level at zero flow is about 0.13 mm/s.

## References

[1] Kuo J.T.W., Yu L., Meng E. (2012). Micromachined thermal flow sensors—A review. Micromachines.

[2] Silvestri S., Schena E. (2012). Micromachined flow sensors in biomedical applications. Micromachines.

[3] (2008). JCGM 200: 2008—International Vocabulary of Metrology Basic and General Concepts and Associated Terms (VIM).

[4] Incropera F.P., DeWitt D.P. (2002). Fundamentals of Heat and Mass Transfer.

[5] Lang W. (1990). Heat transport from a chip. IEEE Trans. Electron. Devices.

[6] Van Putten M.J.A.M., van Putten M.H.P.M., van Putten A.F.P. (1999). Thermal flow measurements at Gr/Re^2^ ≫1 by silicon anemometry. IEEE Trans. Instrum. Meas..

[7] Cubukcu A.S., Zernickel E., Buerklin U., Urban G.A. (2010). A 2D thermal flow sensor with Sub-mW power consumption. Sens. Actuators A Phys..

[8] Buchner R., Bhargava P., Sosna C., Benecke W., Lang W. Thermoelectric Flow Sensors with Monolithically Integrated Channel Structures for Measurements of Very Small Flow Rates.

[9] Patsis G.P., Petropoulos A., Kaltsas G. (2012). Modelling and evaluation of a thermal microfluidic sensor fabricated on plastic substrate. Microsyst. Technol..

[10] Liao S.H., Chen W.J., Lu M.S.C. (2013). A CMOS MEMS capacitive flow sensor for respiratory monitoring. IEEE Sens. J..

[11] Ashauer M., Glosch H., Hedrich F., Hey N., Sandmaier H., Lang W. Thermal Flow Sensor for Liquids and Gases.

[12] Kaanta B.C., Chen H., Zhang X. (2010). Novel device for calibration-free flow rate measurements in micro gas chromatographic systems. J. Micromech. Microeng..

[13] Sun J.-B., Qin M., Huang Q.-A. (2007). Flip-chip packaging for a two-dimensional thermal flow sensor using a copper pillar bump technology. IEEE Sens. J..

[14] Buchner R., Sosna C., Maiwald M., Benecke W., Lang W. (2006). A high temperature thermopile fabrication process for thermal flow sensors. Sens. Actuators A Phys..

[15] Sosna C., Walter T., Lang W. (2011). Response time of thermal flow sensors with air as fluid. Sens. Actuators A Phys..

[16] Issa S., Sturm H., Lang W. (2011). Modeling of the response time of thermal flow sensors. Micromachines.

[17] Kim T.H., Kim D.-K., Kim S.J. (2009). Study of the sensitivity of a thermal flow sensor. Int. J. Heat Mass Trans..

[18] Johnson J.B. (1928). Thermal agitation of electricity in conductors. Phys. Rev..

[19] Kester W. Taking the Mystery out of the Infamous Formula, NR= 6.02 N+ 1.76 dB“and Why You Should Care.

